# Optimizing *Brassica oleracea* L. Breeding Through Somatic Hybridization Using Cytoplasmic Male Sterility (CMS) Lines: From Protoplast Isolation to Plantlet Regeneration

**DOI:** 10.3390/plants13223247

**Published:** 2024-11-19

**Authors:** Miriam Romero-Muñoz, Margarita Pérez-Jiménez

**Affiliations:** Department of Biotechnology, Genomic and Plant Breeding, Institute for Agroenvironmental Research and Development of Murcia (IMIDA), c/Mayor s/n, E-30150 Murcia, Spain; margarita.perez3@carm.es

**Keywords:** micropropagation, protoplast fusion, gene editing, Brassicaceae breeding, cytoplasmic male sterility

## Abstract

The *Brassica oleracea* L. species embrace important horticultural crops, such as broccoli, cauliflower, and cabbage, which are highly valued for their beneficial nutritional effects. However, the complexity of flower emasculation in these species has forced breeders to adopt biotechnological approaches such as somatic hybridization to ease hybrid seed production. Protoplasts entail a versatile tool in plant biotechnology, supporting breeding strategies that involve genome editing and hybridization. This review discusses the use of somatic hybridization in *B. oleracea* L. as a biotechnological method for developing fusion products with desirable agronomic traits, particularly cytoplasmic male sterile (CMS) condition. These CMS lines are critical for implementing a cost-effective, efficient, and reliable system for producing F1 hybrids. We present recent studies on CMS systems in *B. oleracea* L. crops, providing an overview of established models that explain the mechanisms of CMS and fertility restoration. Additionally, we emphasize key insights gained from protoplast fusion applied to *B. oleracea* L. breeding. Key steps including pre-treatments of donor plants, the main tissues used as sources of parental protoplasts, methods for obtaining somatic hybrids and cybrids, and the importance of establishing a reliable plant regeneration method are discussed. Finally, the review explores the incorporation of genome editing technologies, such as CRISPR-Cas9, to introduce multiple agronomic traits in *Brassica* species. This combination of advanced biotechnological tools holds significant promise for enhancing *B. oleracea* breeding programs in the actual climate change context.

## 1. Introduction

**Figure 1:**
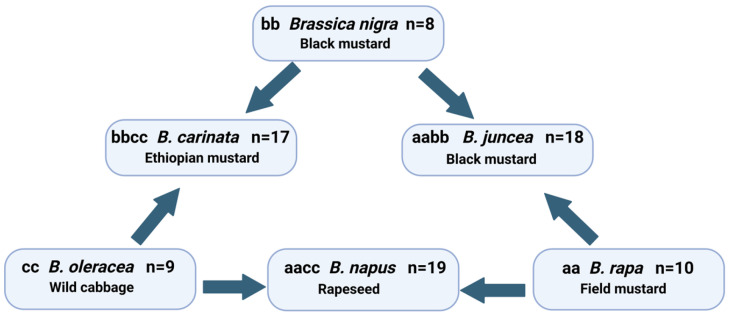
Phylogenetic relationships between species from the Brassica genus (U triangle) as proposed by Nagaharu [4]. The basic number of each species and its chromosomic conformation (n) have been indicated. Letters a, b and c represent the chromosomes of the three diploid species (*B. rapa*, *B. nigra*, and *B. oleracea*) corresponding to the aa, bb, and cc genomes, respectively. The three allotetraploid species (*B. juncea*, *B. napus*, and *B. carinata*) are hybrid combinations of these basic genomes.

The genus *Brassica* belongs to the family *Brassicaceae* (=*Cruciferae*) and comprises a large number of species that are distributed throughout the world due to their adaptability to a wide range of climatic conditions [1]. From an economic point of view, this genus is the most important in the family, as it includes several species that are cultivated for their edible roots, stems, leaves, buds, flowers, spices, oilseeds, and fodder crops [2]. According to Cai et al. [3], this genus is considered a good example of plant domestication due to the large number of species of agronomic interest: broccoli, cauliflower, cabbage and Brussels sprouts (*Brassica oleracea* L.), turnips (*B. rapa* L.), mustard (*B. nigra* L.), etc. From all the species in the genus, the three diploid species *B. nigra* (L.) Koch (genome bb, 2n = 16), *B. oleracea* L. (genome cc, 2n = 18), and *B. rapa* L. (genome aa, 2n = 20) are almost exclusively of economic importance and altogether form the triangle of U proposed in 1935 by Nagaharu [4,5] based on the cytology of the genus and the relationships between the genomes of the different species (Figure 1).

The most widely grown members of the family with different morphogenic appearances of *B. oleracea* encompass cauliflower (var. *botrytis*), cabbage (var. *capitata*), broccoli (var. *italica*), Brussels sprouts (var. *gemmifera*), kohlrabi (*gongylodes*), and kale (var. *sabellica*), and they are sometimes referred to as “cole” crops [3]. Among all of them, broccoli (*B. oleracea* L. var. *italica*), cauliflower (*B. oleracea* L. var. *botrytis*), and cabbage (*B. oleracea* L. var. *capitata*) are considered one of the most economically important cool-season vegetable crops [6] and are known for their pharmaceutical and nutritional properties. These crops owe their popularity to the anticancer bioactive compounds caused to human health, which include isothiocyanates, which are the bioactive compounds obtained by glucosinolates hydrolysis, glucoraphanin, selenium, and flavonoids. Additionally, they are known to be rich in vitamin C, proteins, and minerals and contain the anticancer active ingredient sulforaphane, especially in broccoli, which can significantly reduce the risk of a variety of cancers, cardiovascular and several diseases [7,8,9]. Nowadays, driven by the nutraceutical properties of these vegetables, market demand is increasing. To meet the food needs of a projected population of nine billion people by 2050, an increase in food production of between 70% and 90% will be needed, which will require an increase in the yield of major crops [10]. This increase must be achieved in a sustainable manner, without increasing the demand for resources (arable land, irrigation water, and fertilizers) and without exceeding yield losses due to environmental stresses. While biotic factors (diseases, pests, and weed competition) account for less than 10% of yield losses, abiotic constraints such as drought and salinity, exacerbated by climate change, account for the remaining 70% [11]. *B. oleracea* L. production is being reduced on a global scale by the effects of climate change and a variety of abiotic stresses, such as drought, extreme temperatures, soil salinity, and nutrient deficiencies [12]. These stressors not only reduce overall yields but also cause crop damage in multiple ways, including delayed growth cycles, reduced crown size, leaf wilting, and diminished nutrient content, all of which compromise crop quality and marketability [13,14]. Prolonged drought, in particular, can significantly impact yields by extending the growing season and compromising crown quality [15]. These challenges are driving breeders and researchers in the search for new varieties with traits that improve resilience and adaptability to diverse and changing climatic conditions.

Classical breeding programs initially focused on selecting and crossing existing varieties to produce offspring with improved characteristics, focusing on the breeding of first-generation (F1) hybrids and exploiting additive and dominant effects [16]. Hybridization has been a fundamental tool in *B. oleracea* L. breeding programs, with the aim of producing varieties with specific traits adapted to farmers’ needs and market demands. In addition, F1 hybrids typically show a high degree of uniformity in plant characteristics, including growth habit, maturity, and quality. This increased productivity can be attributed to the combination of complementary traits from the two parent lines, resulting in better overall performance in terms of crop production. Other benefits include resistance to disease and to different environmental conditions and stresses such as heat, drought, or soil conditions, making them more versatile for cultivation in different regions [17,18]. Since the activity of breeders is conditioned by the reproductive systems of the plants, the methods used for genetic improvement of vegetable brassicas have been those typical of cross-pollinated plants. In cross-pollinated species such as *B. oleracea* L., pure lines can be obtained by forced self-pollination, and the crossing of two pure lines will produce a hybrid with vigor and production generally superior to that of the parent lines. Using sterile lines to produce hybrid seeds can reduce seed production costs and improve the purity of hybrid seeds, which is an ideal approach to harness heterosis [19]. This method allows for hybrid seed production without the risk of hybrid plants setting seed, ensuring that desirable traits are preserved while preventing unwanted genetic mixing. However, the difficulty in obtaining F1 hybrid seeds is due to the flower morphology of the Brassicaceae crop species. *B. oleracea* flowers are hermaphroditic and typically bilaterally symmetrical, arranged in corymb-like inflorescences that generally lack bracts. The androecium consists of six stamens, with two shorter than the others, while the gynoecium is formed by two fused carpels, creating a superior ovary. Moreover, these flowers exhibit protogyny, meaning the female parts mature before the male parts, facilitating cross-pollination [20]. Hence, *B. oleracea* L. crops are cross-pollinated, and their pollination is favored by self-incompatibility, which means that the pollen grains must come from other plants. Because of challenges related to sexual incompatibility, traditional plant breeding is a time-consuming process, often taking 8–10 years to develop a new variety, making it a very long process for crop genetic improvement [14]. In *B. oleracea*, the commercial production of F1 hybrids generally relies on self-incompatibility systems, which are genetically intricate and difficult to breed and select. This problem has been overcome by various advances in in vitro techniques such as somatic hybridization or protoplast fusion, which have offered powerful tools to address this challenge using cytoplasmic male sterile (CMS) lines. The CMS trait would simplify the breeding of parental lines and the production of hybrid seeds [21]. Hybrid production using CMS lines has become an important way of exploiting heterosis in vegetables, since CMS eliminates the need for anther removal, promoting hybrid technology to produce significantly enhanced F1 progeny. These hybrids outperform their parent lines and popular existing cultivars in areas such as yield, stress tolerance, adaptability, etc. [22]. Somatic cell fusion techniques show great potential for developing innovative crop varieties capable of meeting the demands of a growing population while also adapting to the challenges of climate change [23]. Therefore, the availability of male sterile lines with interesting agronomic traits is an important factor in enhancing the yield and quality of hybrid seeds in the Brassicaceae family.

Considering the crucial role that crop hybrids play in contemporary food production systems, employing CMS-based hybrid technology via somatic hybridization offers a promising strategy for sustainably enhancing crop yields in Brassica breeding [24]. This review begins with a retrospective look at the discovery of diverse sterilizing cytoplasms in Brassica, focusing on the candidate genes responsible for the CMS condition and the production of male sterile genotypes through protoplast fusion. We place emphasis on recent insights gained from protoplast fusion applied to *B. oleracea* in developing CMS-based lines. Critical steps include the pre-treatments of donor plants, the selection of different tissues as sources of parental protoplasts, the techniques for generating somatic hybrids and cybrids, and effective plant regeneration methods. Additionally, we underscore the potential to integrate multiple valuable agronomic traits through protoplast fusion and CRISPR-Cas genome editing technology in *B. oleracea* species. This combination of advanced biotechnological tools offers a powerful strategy for enhancing Brassica breeding programs and improving crop productivity.

## 2. Methodology of Research

A comprehensive literature review was conducted through searches in Scopus, ScienceDirect, Google Scholar, NCBI, and Springer databases using keywords such as “Brassica” or “Brassica oleracea”, “protoplast regeneration” or “protoplast fusion”, “in vitro” or “tissue culture”, “crop improvement”, “plant breeding”, “cytoplasmic male sterility”, and “gene editing” or “CRISPR/Cas”. This search aimed to capture current research trends in the field of protoplast regeneration. Of the initial 689 articles identified on topics including protoplast regeneration, genomic transformation, and cytoplasmic male sterility, a thorough screening process based on titles, abstracts, and full texts reduced the set to 354 relevant studies, of which 169 were selected for further analysis. These studies span key areas such as cytoplasmic male sterility in the Brassicaceae family (with a focus on 43 studies), isolation protocols (22 studies), protoplast fusion procedures (39 studies), regeneration through direct and indirect organogenesis (26 studies), and plant transformation through genetic engineering (39 studies). This overview underscores significant research areas in *B. oleracea* crop improvement, particularly highlighting the role of protoplast fusion, gene editing, and tissue culture techniques as vital tools for advancing breeding strategies.

## 3. Cytoplasmic Male Sterility in Brassicaceae Breeding

### 3.1. Discovery of Sterilizing Cytoplasms in Brassicas

The phenomenon of male sterility (MS) was first observed in 1873 by the studies on plant fertility in hybrid individuals carried out by the German botanist Joseph Gottlieb Kolreuter, in which he reported on anther abortion within species and specific hybrids. Thus, researchers could define MS as the failure of plants to produce functional pollen, dehiscent anthers, or viable male gametes [25]. This leads to an abnormal development of either the sporophytic or gametophytic anther tissues in MS individuals [26]. Up to now, MS is a common trait observed in 43 families, 162 genera, and approximately 617 species, including *Allium cepa*, *Glycine max*, *Gossypium hirsutum*, *Raphanus sativus*, *Sorghum bicolor*, *Oryza sativa*, and *Zea mays* (reviewed by [27]). Regarding the classification of male sterility (MS), two main types are distinguished based on inheritance characteristics: genic male sterility (GMS) and cytoplasmic male sterility (CMS). The key difference between these types lies in their genetic control. GMS is governed by nuclear genes and can be maintained by crossing with fertile lines [28,29]. Whereas CMS, including cytoplasmic genic male sterility (CGMS), is primarily controlled by mitochondrial genes, with a smaller contribution from nuclear genes, resulting in maternal inheritance [30,31]. The introduction of MS into a crop is typically achieved through inter- or intraspecific crosses, though it can also arise from mutations in mitochondrial open reading frames (ORFs). In contrast to CMS, most GMS mutants are not suitable for hybrid seed production, as maintaining MS efficiency in these lines can be challenging [32].

CMS arises from incompatibilities between the nuclear genome and foreign mitochondrial genomes, a phenomenon referred to as alloplasmic communication. This condition is caused by rearrangements in mitochondrial DNA (mtDNA) and has been documented in over 140 plant species, including various Brassicaceae horticultural species [33]. In the Brassica genus, multiple CMS systems have been identified, with the Ogura CMS system [34]—first discovered in Japanese radish (*Raphanus sativus*)—being the most extensively studied among Brassicaceae plants [34]. Additionally, other CMS sources have been developed, such as the Nap CMS system [35], Tour CMS [36], Nig CMS [37], Polima (Pol) CMS [38,39], Lembkand male-sterile (MSL) CMS [40,41], and Hau CMS [42].

Amongst all of them, Ogura CMS has demonstrated environmental stability in maintaining sterility and generally exhibits fewer floral abnormalities compared to others. After its discovery in Japanese radish, the sterile cytoplasm from Ogura was successfully transferred to several Brassica and radish cultivars through a backcrossing approach [27]. As a result, Ogura CMS has become the most widely adopted CMS system in cruciferous crops due to its significant advantages of stable sterility and complete stamen abortion. In broccoli and kale, successive backcrosses effectively facilitated the transfer of Ogura CMS, and later, from broccoli to cauliflower [43]. Kamiński [44] identified and selected stable Ogura-INRA from Raphanus sativus, which was then introduced into three broccoli lines for breeding Ogura CMS broccoli. Ogura cytoplasm is widely employed in hybrid breeding programs for *B. napus* and *B. juncea* [27]. Furthermore, the pol CMS in *B. napus* stands as another well-recognized example of spontaneous male sterility [45]. In *B. juncea*, both sexual and somatic hybridization with wild relatives have resulted in the development of several CMS lines [46]. Mitochondrial markers can be used to quickly and accurately distinguish between the different types of CMS sources [47]. The study by Shu et al. [30], which was conducted in broccoli, used mitochondrial markers to analyze the diversity of CMS sources in 39 broccoli accessions, including 19 CMS lines and 20 hybrids, and determined that all CMS accessions contained the Ogu orf138-related DNA fragment. For all aforementioned, it can be concluded that Ogura CMS has gradually become a widely used cytoplasmic sterility vector in *Brassica oleracea* L. hybrid seed production.

### 3.2. Three-Line CMS/Rf Breeding System

**Figure 2:**
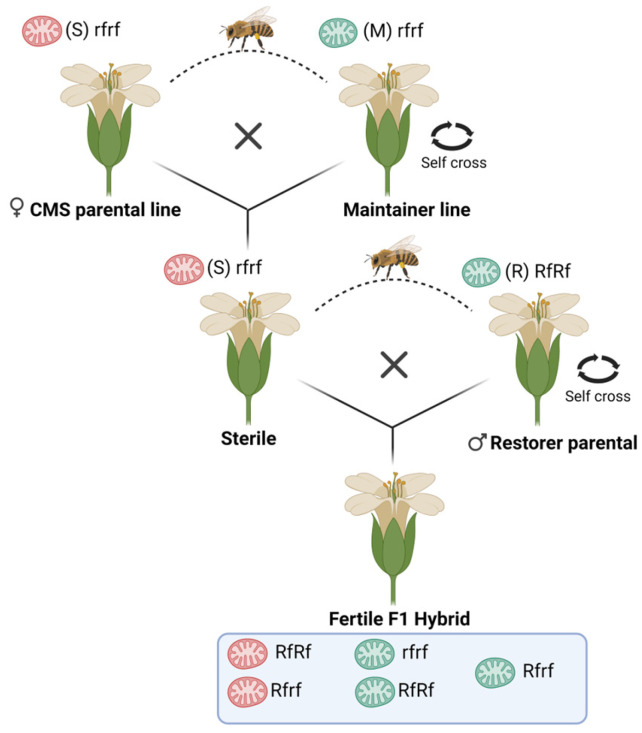
Diagram of the three-line system used in cytoplasmic male sterility (CMS) hybrid seed production. The system includes a CMS line (S) with sterile cytoplasm and a non-functional restorer gene (rf), a maintainer line (M) with normal cytoplasm and an identical nuclear genome to the CMS line, and a restorer line (R) with normal or sterile cytoplasm and functional restorer gene(s) (Rf). The CMS line is propagated by crossing with the maintainer line, while both the maintainer and restorer lines can self-pollinate to produce seeds. Male fertile hybrids are produced by crossing the CMS line with the restorer line. Adapted from [62].

Following Chen and Liu [48], the CMS-based hybrid seed production system involves three key breeding lines: the CMS line (S), the maintainer line (M), and the restorer line (R). The CMS line carries male-sterile cytoplasm and a gene responsible for sterility (CMS gene) but lacks a functional restorer of fertility (Rf) gene. This line is used as the female parent in hybrid crosses. The maintainer line, which contains normal fertile cytoplasm and shares the same nuclear genome as the CMS line, is used as the male parent to maintain the CMS line through backcrossing. The restorer line, which carries one or more Rf genes, serves as the male parent in crosses with the CMS line to produce hybrid F1 seeds. The restorer line is crucial for restoring fertility in the CMS line, as the only difference between the S and M lines is the sterility-inducing cytoplasm (Figure 2). In some wild plant species, CMS cytoplasm exists alongside male fertility due to the presence of Rf genes in their nuclear genomes. Consequently, CMS cytoplasm is usually identified through genetic crosses or somatic hybridization, which enables the separation of the CMS cytoplasm from the associated Rf gene(s) in the nuclear genome [49]. In the F1 generation, the Rf gene restores fertility, and the combination of nuclear genomes from the CMS and restorer lines leads to the expression of hybrid vigor, resulting in improved performance in the F1 hybrids produced by the S × R cross [50].

Typically, nuclear restorers are provided by species that serve as cytoplasmic donors [46,51]. However, Ogura CMS restorers are absent in *B. oleracea* cultivars, with all fertile forms acting as maintainers [52]. Research on the restorer line for Ogura CMS from *B. napus* has been ongoing for more than 30 years [53,54]. Rf has been transferred into *B. napus* from radish, after which it was integrated into chromosome C09 of *B. napus* [55], which encoded the creation of the restorer line successfully, and it was applied to the production of Ogura CMS seeds of *B. napus*. The Ogura CMS/Rf breeding system has established itself as a key technique for hybrid production in *B. napus*. Consequently, *B. napus* can act as an Rf gene donor to enhance oilseed rape cultivars [56,57,58,59], resulting in the development and widespread distribution of numerous exotic cultivars around the globe [60]. However, cross-incompatibility poses challenges to natural hybridization between distant relatives, such as species within *B. oleracea*. To overcome these obstacles, methods including sexual hybridization, multiple pollinations, and embryo rescue are utilized to increase the chances of success in interspecific crosses between *B. napus* and broccoli, with the goal of creating CMS broccoli lines. The CMS technology has emerged as a significant asset for harnessing hybridization techniques in crop plants due to its unique capability to produce sterile male gametophytes while maintaining the agronomic performance of the plants [24,61].

### 3.3. Molecular Mechanism of CMS/Rf System in Brassicas

**Figure 3:**
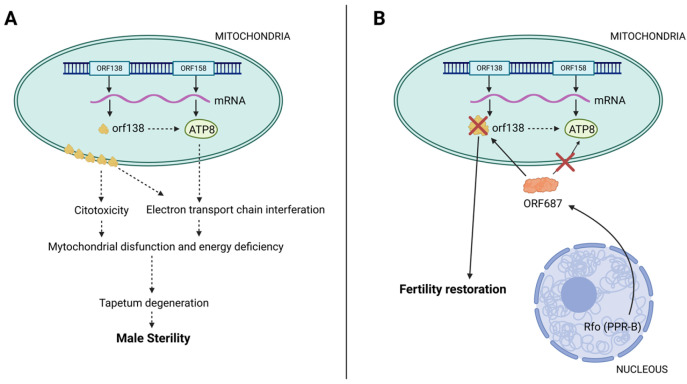
Schematic overview of the molecular mechanisms underlying (**A**) the cytoplasmic male sterility (CMS) system and (**B**) the fertility restoration process in Brassicas.

The CMS/Rf system is the most prevalent male sterility system used in cruciferous crops [49]. This system can be described as a genetic model involving two genetic components, each with distinct inheritance patterns, one at the cytoplasmic level and the other at the nuclear level [63]. In interspecific crosses, cytoplasmic male sterility is often caused by the interaction between the cytoplasm of one parent and the nuclear genome of the other parent [64]. In brassicas, the mitochondrial genes responsible for androsterility are the orf138 gene encoding the ORF138 protein and the orf158 gene encoding the ATP8 protein [27,65]. Overexpression of orf138 results in the accumulation of ORF138 protein in the mitochondrial membrane, leading to cytotoxicity. On the other hand, ATP8 protein interferes with the electron transport chain, which together with the action of ORF138 leads to dysfunction of mitochondrial function and energy deficiency [66]. Fertility restoration involves the Rfo genes found in nuclear DNA (Figure 3). In brassicas, the restoration of fertility is mediated by the Rfo gene, which encodes the PPR-B protein, also referred to as ORF687 [67]. Although the levels of orf138 mRNA in the anthers do not change, the accumulation of ORF138 is notably suppressed. PPR-B interacts with orf138 mRNA, likely blocking the translation of ORF138 and thereby playing a crucial role in regulating fertility. This interaction helps prevent mitochondrial dysfunction, allowing the anthers to develop normally and restoring male fertility. Mitochondrial sorting gene (MSG) products encoded in the nucleus—including Rf proteins and tissue-specific regulatory factors (TSRFs) and mitochondrial sorting gene subunits (TSRFs) and mitochondrial electron transfer chain subunits (mtETCs)—are targeted to mitochondria for upstream regulation. According to Chen and Liu [48], TSRFs can modulate the accumulation of CMS proteins (such as ORF138 in brassicas) in a male organ (anther)-specific manner, operating at either the translational or post-translational level to influence male specificity. These CMS proteins have the ability to interact with subunits of the mtETC, which can affect their functions, redox state, or ATP biogenesis. This interaction results in the production of retrograde signals, including reactive oxygen species and cytochrome c release, which can trigger abnormal programmed cell death (PCD) in the tapetum. As previously noted, these detrimental effects of mitochondrial proteins lead to CMS phenotypes. Therefore, any mechanism that suppresses the expression of CMS genes or counteracts their adverse effects is likely to restore male fertility within CMS/Rf systems. Typically, the species that serve as cytoplasmic donors for sterility also provide nuclear restorers [52,56]. In *B. oleracea* species, Ogura sterility is characterized by the absence of fertility restorer genes, which means that all fertile forms act exclusively as maintainers [44]. The first male sterile line of broccoli was identified through a homologous comparative analysis using BLAST in GenBank. This analysis revealed a 100% homology in specific segments of three sterility materials (LI-Cl, LI l-CI, and L3-FI) with the Ogu orf138 gene (GenBank accession No. HQ149728) associated with the reported broccoli Ogu CMS [31].

## 4. Use of Protoplast Technology in *Brassica oleracea* L. Crops

### 4.1. Discovering Protoplasts Technology

The term protoplast was first coined by Harnstein in 1880 to designate the naked plant cells without their cell wall. The first protoplast isolation was achieved by Klercker in 1892 using mechanical methods to obtain protoplasts from the water plant *Stratiotes aloides* [62]. However, it was not until 1965 when Gregory and Cocking discovered the enzymatic method to isolate the protoplast from the parenchymatous placental tissue of immature tomato fruit using crude extracts with cellulase activity obtained from the fungus *Myrothecium varrucana* [68]. Once established the efficacy of the enzymatic method, the technique has been subject to multiple refinements and adjustments, being applied to a multitude of plant species. Henceforth, due to plant cell totipotency, direct plant regeneration from mesophyll cell protoplasts has been successfully achieved in a significant number of dicotyledonous species [69]. This demonstrates that protoplasts can re-initiate cell division and form a callus, thereby enabling the potential for plant regeneration [70]. In early reports, Shepard and Tottem [71] were successful in achieving the whole plant regeneration from tobacco (*Nicotiana tabacum* L.) protoplasts. Otherwise, one of the earliest demonstrations of somatic embryogenesis was reported by Dudits et al. in 1976 from carrot protoplasts [72]. In *B. oleracea* L., the first somatic embryogenesis was reported by Pareek and Chandra in 1978 from the leaf callus of cauliflower (*B. oleracea* var. *botrytis*) [73]. Shortly thereafter, during the 1980s, rapid progress occurred in protoplast fusion to improve plant genetic material, the development of transgenic plants, and plant breeding [74].

Protoplast fusion from different species and/or varieties is a process known as somatic hybridization and can generate new varieties with the combined characteristics of the parents used. This approach is used in plant breeding to develop strains that possess desired agronomic traits such as disease resistance [59], tolerance to adverse climatic conditions [75], male sterility [21,51,76] and other valuable characteristics. The Brassica genus has made it possible to obtain interspecific and intergeneric somatic hybrids with special nuclear and cytoplasmic genetic combinations (e.g., cytoplasmic androsterility or cybridity), obtaining cytoplasmic hybrids or cybrids, by transferring extrachromosomal genes that confer special characteristics to the fused cells. One of the most relevant hybridization model cases reported is Brassica–Raphanus. In this experiment conducted by Jourdan et al. in 1989 [77], the heterokaryon contained the nucleus of *B. napus* (rapeseed), the atrazine-resistant chloroplasts of *B. campestris* (field mustard), and the mitochondria of *R. sativus* (radish), which bestow androsterility. After the discovery of orf138 of Ogura CMS in *R. sativus* the sterile cytoplasm was transferred to *B. oleracea* species using sexual and somatic hybridization with wild relatives to deliver diverse CMS lines [49]. This fact caused the breeding of the modern CMS *B. oleracea* F1 cultivars as cauliflower, broccoli, and cabbage as well as for rapeseed F1 hybrids [31,51,78].

All in all, protoplast fusion has been the main procedure to generate CMS condition de novo in Brassicas since somatic hybridization prevents the unwanted or uncontrolled transmission of traits that may arise from the simultaneous inheritance of genes other than those responsible for CMS. This finding has led to immense progress in somatic cell genetic engineering by allowing the hybridization of incompatibility genetic material into cells without the cell wall and has become an important tool for research in fundamental and applied biology studies as well as in breeding improvement processes. Nevertheless, the successful use of somatic hybridization in this context requires a number of prerequisites. These involve the efficient and reliable isolation of large quantities of highly viable protoplasts from both partners, as well as establishing reproducible strategies that facilitate high-frequency plant regeneration from the cultured protoplasts of at least one of the potential fusion partners. Additionally, it is important to develop effective methods for obtaining viable heterokaryons following protoplast fusion, ensuring these heterokaryons can successfully undergo division and ultimately lead to plant regeneration. The information available hitherto on these critical steps as tissue sources, fusion procedures, and in vitro culture methods to obtain plants from protoplast isolation for the *B. oleracea* L. members is discussed in the following sections.

### 4.2. Tissue Sources and Cell Wall Digestion Procedures for Protoplast Isolation

A reliable source of good-quality protoplast is needed to achieve plant regeneration successfully. Different source materials used for protoplast isolation can significantly affect the number, size, viability, and regenerative abilities of the resulting protoplasts. To date, a variety of explants have been used to isolate protoplasts in *B. oleracea* L. species such as broccoli, cauliflower, or cabbage. These reports (see Reference Table 1) involve numerous tissues, which include hypocotyls, cotyledons, leaves, callus, and cell suspension cultures, and all of them have been used as a source of viable protoplasts and tested for their ability to regenerate plants. According to Pasternak et al. [69], cotyledon protoplasts offer a significant advantage due to the relative uniformity of the starting material, providing the potential to achieve various levels of differentiation. However, cotyledons can only be utilized as a protoplast source before entering the endocycles, as the rapid differentiation process in cotyledons leads to an increase in nuclear DNA content and a high level of chromatin condensation. In brassicas, cotyledons are one of the most used tissues for protoplast isolation, reaching incredibly high levels of viability (see Reference Table 1). Another popular source of protoplast in brassicas is the use of hypocotyls, which can be isolated from dark-grown seedlings and share similar benefits with cotyledon protoplasts, as they generate a homogeneous and synchronized cell population that results in enhanced plantlet regeneration [79]. As examples can be cited the studies carried out on *B. oleracea* var. *italica* [80,81], var. *capitata* [82,83,84], and var. *botytris* [80,85]. However, a further disadvantage of these sources, both hypocotyl and cotyledons, is the large number of seeds needed. This fact entails the use of true leaves as the most commonly used source of protoplasts in the Brassica genus, although true leaves exhibit varying biological ages and developmental stages, which produce different capacities for cell differentiation [69]. The latter option usually used as a source for viable protoplast in *B. oleracea* L. is the use of callus; however, this source is not as exploited as the previous ones mainly due to the time-demanding and low concentrations of protoplast.

Apart from all the above-mentioned, the status of the donor plant cells is one of the main critical factors to achieve plantlet regeneration [86]. The plant material routinely used as source protoplast tissues could be picked up from plants grown in the field, in a greenhouse, as well as plants grown aseptically in vitro. Similarly, different tissues provide several limitations and advantages; the environment in which a plant is grown also affects the viability of the isolated protoplast and entails a critical step to reach successful plant regeneration. Greenhouse cultures are commonly accessible; however, they rely on temperature, light, and nutrient availability, which are not always adequately controlled for optimal growth. Thus, protoplasts can be isolated from micropropagated clones cultivated under controlled conditions, minimizing the variability associated with plants grown in greenhouses and fields [87], which reduces the negative effects of suboptimal physiological conditions on isolation efficiency. In *B. oleracea*, the age of the explant and the choice of tissue source are among the most crucial factors for protoplast isolation. Leaf explants aged 4 to 6 weeks and hypocotyls that are 1 week old demonstrated significantly higher protoplast viability compared to 2-week-old tissues from either source [84].

To achieve successful isolation of the protoplast, essential knowledge of the cell wall composition is required. Plant cell walls are composed of mesophyll, primary, and/or secondary cell walls. Generally, it can be considered that cell walls are mainly composed of polysaccharides (cellulose, hemicellulose, and pectin), which constitute over 90% of the primary cell wall dry weight and 65–85% of the secondary cell wall dry weight [88,89]. The middle lamella is mainly composed of pectins and hemicellulases, while the primary cell walls are composed of cellulose, hemicelluloses, pectins, and structural proteins. The polysaccharide composition of primary cell walls differs between monocots and dicots [82,88]. Monocotyledons have a primary cell wall primarily composed of arabinoxylans and cellulose, while in dicotyledons, cellulose, pectins, and xyloglucans are the dominant polysaccharides. Furthermore, secondary cell walls differ from primary cell walls, which mainly consist of xylans and cellulose. The deposition of secondary cell walls is also marked by the incorporation of lignin, which enhances the structural integrity of the cell walls. Also, the matrix composition of polysaccharides and structural proteins is highly variable between species and cell types [90]. Owing to the different polysaccharide composition of the cell wall, enzyme mixture entails another important factor for protoplast isolation. Enzymes can be classified into two groups, namely, pectinases, which dissolve the middle lamella and separate individual cells, and cellulases and hemicellulases, which decompound the cell wall and release the protoplast.

To date, various enzyme mixtures and combinations of different concentrations have been used to obtain better protoplast yield (see References Table 1). Commonly, Cellulase Onozuka RS, Pectolyase Y-23, and Macerozyme R10 are used together for the isolation of protoplasts. However, the concentration and enzyme mixture depend on the species in question. In the case of Brassicas, several studies studied cell wall compounds, and they stated that cellulose and xylans were the most abundant polysaccharides in most *B. oleracea* L. culture species such as broccoli, cauliflower, or kohlrabi [91,92,93]. In *B. oleracea* L., protoplasts are routinely isolated from leaves and hypocotyls using various enzyme solutions. Typically, Cellulase Onozuka RS is employed at concentrations ranging from 0.1% to 1.5%, often in combination with 0.1% to 1% Macerozyme R10 or 0.1% Pectolyase Y-23. (see References in Table 1). Several studies have reported lower yields (1.8 × 10^4^ to 0.8 × 10^6^ protoplasts g^−1^ FW) for hypocotyls than for leaves (1.3 to 3.2 × 10^6^ protoplasts g^−1^ FW) [84,94,95], though this latter is a good source of obtaining a high yield of uniform protoplast. Moreover, in cabbage (*B. oleracea* var. *capitata* L.), it was observed that protoplasts derived from hypocotyls produced more regenerated shoots compared to those derived from leaves [82]. Additionally, the recent study of Stajič [79] carried out in cabbage tested the quantity and quality of isolated protoplasts using different concentrations of Pectolyase Y-23. Their study revealed that the increase in enzyme concentration resulted in higher yields, but high enzyme concentrations can result in phytotoxicity, which subsequently decreases protoplast viability. Moreover, the addition of Ca^2+^ ions to enzyme solution and organic buffers such as morpholinoethane sulphonic acid (MES) increases the stability of protoplasts by minimizing the changes in pH during the incubation time. Otherwise, the time of action of the enzyme solution varies from 12 to 18 h in the majority of the isolation protocols (see References Table 2). The application of pre-treatments to the source tissues prior to enzyme digestion is also advised to ensure the successful isolation of viable protoplasts. These treatments may consist of physical disruption techniques, such as chopping the tissues or removing the epidermis, as well as a pre-plasmolysis step that causes the protoplasts to shrink away from the cell wall prior to the introduction of the enzyme solution [70,96]. Pre-plasmolysis solution can contain either sucrose or mannitol, which is often preferred in most protocols of Brassicas protoplast isolation [97].

### 4.3. Culture Media Formulation and Plant Regeneration

The de novo regeneration of the cell wall after protoplast isolation is a crucial step for the effective use of protoplast technology in plant breeding. Among all factors influencing protoplast regeneration, plant growth regulators (PGRs) are considered the most crucial. The general understanding is that an elevated auxin-to-cytokinin ratio effectively promotes cell division and cell wall formation in protoplasts, while a higher cytokinin-to-auxin ratio is crucial for shoot regeneration [98]. Nevertheless, the optimal ratio can differ considerably between different species [70]. This fundamental stage has been extensively studied in various horticultural species, including Brassicaceae cultivars (see references Table 2). In an earlier study, Quazi [99] examined the structure of the cell wall regenerated by *B. oleracea* L. protoplasts from different cultivars to better understand the mechanisms that enhance their viability and division competence during culture. This study revealed that cell wall regeneration is a prerequisite for cytokinesis. Additionally, other researchers isolated protoplasts from the cotyledons of several cultivars of *B. napus*, *B. campestris*, and *B. oleracea*, and cultured them in different media to study the characteristics of cell wall regeneration and cell division during the early stages of culture. They found that cell wall regeneration begins 2–4 h after isolation [100,101]. Furthermore, it was reported that while the cell wall regeneration in protoplast culture was not affected by the culture medium used, the composition of the culture medium plays a significant role in determining the level of cell division, which is directly linked to the plant’s regeneration capacity. Soon after these studies, Emmerling and Seitz [102] isolated a xyloglucan oligosaccharide from the cell walls of suspension-cultured carrot cells and studied its effects on regenerating carrot protoplasts. This nonasaccharide, designated XG9 (Glc4Xyl3GalFuc), exhibited anti-auxin characteristics. The introduction of XG9 in nanomolar concentrations to media containing 2,4-Dichlorophenoxyacetic acid (2,4-D) impacted both the viability of the isolated protoplasts and the activity of glycan synthases, producing similar effects to those observed when 2,4-D was excluded from the regeneration medium. This finding aligns with a previous report that indicated 2,4-D was essential for cell wall formation and initial protoplast growth [103]. Several researchers have utilized high concentrations of 6-benzylaminopurine (BAP) in combination with 1-naphthaleneacetic acid (NAA) for shoot regeneration in the Brassicaceae family [104,105]. However, recently the study of Li et al. [98] found that the use of high thidiazuron (TDZ) concentrations gave the best shoot regeneration in comparison with BAP and zeatin concentrations. Moreover, they reported that a high concentration of TDZ in combination with a relatively high level of NAA had a great positive effect on protoplast regeneration in *B. napus*. Interestingly, the study summarized that although BAP is widely used for many crops for in vitro cultures, it did not seem to be effective for protoplast regeneration in *B. napus*. Robertson and Earle [106] successfully achieved plant regeneration from mesophyll protoplasts of *B. oleracea* L. var. *italica*. The protoplasts were isolated from the leaves of the F1 broccoli cultivar Green Comet using an enzyme combination of 2% *w*/*v* cellulose R-10, 1% *w*/*v* Macerozyme R-10, and 0.5% *w*/*v* Driselase, followed by centrifugation to recover the isolated cells. The protoplasts’ totipotency from plants regenerated via hypocotyl explants was superior to that of protoplasts from seedlings. This finding aligns with the work of Zhong and Li [107] and Zhao et al. [100], who also reported successful organogenesis from hypocotyl and cotyledon protoplast cultures in broccoli. Additionally, Mix and Wang [108] described the regeneration of germ-free broccoli through in vitro bud culture, achieving a maximum shoot regeneration rate of 62.5% and an average of 4.1 shoots per explant. In Brassicaceae species, shoot regeneration from protoplasts typically requires low auxin concentrations and elevated cytokinin levels [109]. However, Kiełkowska and Adamus [84] utilized cytokinin alone or a medium completely lacking growth regulators. Otherwise, Stajič [79] demonstrated that adding 0.2 mg/L NAA auxin (instead of just cytokinin BAP) significantly improved shoot formation in cabbage protoplast cultures. The addition of several compounds that enhance cell division and microcallus formation to the culture medium is a common practice to achieve successful plant regeneration.

Recently, the use of phytosulfokine (PSK) in protoplast cultures has been demonstrated to be an important growth factor because of its effect on growth and cell proliferation [83,98]. PSK is a plant-specific disulfated pentapeptide that plays a crucial role in the early stages of cellular dedifferentiation, proliferation, and redifferentiation. When applied at nanomolar concentrations, it behaves similarly to plant hormones and has been demonstrated to improve plant regeneration efficiency through somatic embryogenesis, a process associated with the thinning of cell walls. Godel-Jędrychowska et al. [110] studied the relation between cell wall fractions involved in the regeneration of protoplast-derived carrot cells along with the inclusion of PSK-α in the culture medium. They observed a variable response to PSK-α in protoplast-derived cell development among the three Daucus taxa studied, as well as differences in the chemical composition of the cell walls between the control cultures and those treated with PSK-α [98]. Moreover, Kielkowska and Adamus [83] recently reported that the supplementation of regeneration media with 0.1 µM PSK-α significantly increased broccoli shoot regeneration compared to those grown in media without PSK-α. Furthermore, enhanced regeneration was observed in callus explants derived from leaves of broccoli protoplast treated with PSK-α at the early stages of development and later transferred for regeneration to media supplemented with 0.1 µM of this growth factor.

Apart from the use of PGR in order to control cell division and plant development steps, one of the main disadvantages, in order to achieve plant regeneration from protoplasts derived from cell wall digestion with enzyme preparations, is their high vacuolization and their fragility, hence being difficult to culture. To deal with it, the immobilization of protoplasts has been employed, involving the entrapment of cells in a polymerizing matrix. This method is relatively simple and cost-effective, providing protection for isolated cells and helping to maintain the culture. Numerous studies have suggested that immobilizing protoplasts in a semisolid matrix, such as calcium alginate or agarose disks, facilitates the undisturbed reconstruction of the cell wall, prevents cell agglutination, and promotes the completion of the mitotic division [111,112,113,114]. The study of Kielkowska and Adamus [84] reported for the first time an innovative protocol for the immobilization of *B. oleracea* protoplasts in alginate layers. In that study, mesophyll- and hypocotyl-derived protoplasts from several cabbage cultivars were successfully isolated with satisfactory yields, viability, and division frequencies, enabling their use in further research.

All things considered, tissue culture has become an important platform for all genetic transformation in plant systems [115]. An essential prerequisite for genetic transformation in plants is the development of an effective regeneration protocol. Importantly, the success in protoplast cultures and their subsequent tissue differentiation relies on the presence of genotypic constitutions containing major high-response genes in the available materials or the introgression of these genes into recalcitrant varieties [115,116,117]. Differentiation in in vitro cultures is generally recognized as being genotype-specific, so several efforts in the proper selection of PGRs are critical to achieving plant regeneration. To date, numerous methodologies for regenerating plants from protoplasts have been developed across different species. These techniques offer promising applications in breeding, including the enhancement of genetic diversity through somaclonal variation, the transfer of cytoplasmic or nuclear genes via somatic hybridization, and directly introducing foreign genes through protoplast transformation [29,118]. Within this context, protoplast fusion and somatic hybridization aimed at producing novel hybrids should prioritize desirable agricultural traits, focusing on combinations that can only be achieved through protoplast fusion. These somatic hybrids can then be used in conventional breeding to expand the range of crops where protoplast technology is applicable [119]. However, this approach limits the potential use of protoplasts with specific traits introduced through genetic transformation in fusion experiments.

**Table 1:** Sources for protoplast and isolation conditions in the main *B. oleracea* L. cultivars.

*B. oleracea* L. Cultivars	Tissue Source	Age of Explant	Enzyme Mixture and Condition	Incubation Time	Yield (Protoplast g^−1^ FW)	References
*B. oleracea* var. *italica*	Cotyledons and Leaves	3–4 wk	1.5% Cellulase R-10, 0.4% Macerozyme R-10, 400 mM D-Mannitol, 0.1% BSA, 8 mM CaCl_2_·2H_2_O, 5 mM MES, and 5 mM KOH.	12–16 h	9.1 × 10^6^ g^−1^ FW	[80]
	Cotyledons	7–10 d	0.5% Cellulase R-10, 0.1% Pectolyase Y-23, and 0.6 M D-Mannitol.	12 h	5.05 × 10^6^ g^−1^ FW	[81]
	Leaves	3 wk	1.5% (*w*/*v*) Cellulase R-10, 0.75% *w*/*v*) Macerocyme R-10, 10 mM MES, 0.6 M D-mannitol, 10 mM CaCl_2_·2H_2_O, and 0.1% *w*/*v*) BSA.	3 h	4.2 × 10^6^ g^−1^ FW	[120]
	Leaves	20 d	1.5% Cellulase R-10, 0.4% Macerozyme R-10, 400 mM D-Mannitol, 0.1% BSA, 8 mM CaCl_2_·2H_2_O, 5 mM MES, and 5 mM KOH.	12 h	NR	[121]
		3 wk	2% Cellulysin, 1% Macerozyme R-10, 0.5% Driselase^®^, 0.08 M CaCl_2_, 0.2 M D-Mannitol, 1 mM MES, and 1 mM KH_2_PO_4_.	16 h	1 to 5 × 10^6^ g^−1^ FW	[106]
*B. oleracea* var. *capitata*	Leaves (1) and Hypocotyls (2)	2–6 wk 1–2 wk	(1) 1.0% Cellulase R-10, 0.1% Pectolyase Y-23, 0.6 M D-Mannitol, 20 mM MES, and 5.0 mM MgCl_2_·6H_2_O. (2) 1.0% Cellulose R-10, 0.5% Driselase^®^, 1.0% Macerozyme R-10, 0.6 M D-Mannitol, 20 mM MES, and 5.0 mM MgCl_2_·6H_2_O.	16–18 h	2.0 × 10^6^ g^−1^ FW	[84]
	Leaves	4 wk	0.5% Cellulase Onozuka RS, 0.01–0.1% Pectolyase Y-23 or 0.1% Macerozyme R-10, 2 mM MES, 3 mM CaCl_2_, and 0.4 M D-Mannitol.	16 h	3.75 to 2.75 × 10^6^ g^−1^ FW	[122]
	Cotyledons and Leaves	NR	1.5% Cellulase R-10, 0.4% Macerozyme R-10, 400 mM D-Mannitol, 0.1% BSA, 8 mM CaCl_2_·2H_2_O, 5 mM MES, and 5 mM KOH.	12–16 h	7.0 × 10^6^ g^−1^ FW	[79]
	Hypocotyls	3–4 wk	0.5% Cellulase R-10, 0.1% Pectolyase Y-23, 2 mM MES, 3 mM CaCl_2_, and 0.4 M D-Mannitol.	18 h	NR	[80]
*B. oleracea* var. *botytris*	Cotyledons and Leaves	3–4 wk	1.5% Cellulase R-10, 0.4% Macerozyme R-10, 400 mM Mannitol, 0.1% BSA, 8 mM CaCl_2_·2H_2_O, 5 mM MES, and 5 mM KOH.	12–16 h	7.5 × 10^6^ g^−1^ FW	[80]
	Hypocotyls	3 wk	1.0% Cellulase-R-10, 0.1% Macerozyme R-10, 0.5 M D-mannitol, and 0.55 mM CaCl_2_·2H_2_O.	NR	NR	[85]

Abbreviations used: BSA (bovine serum albumin); MES (2-(N-morpholino)ethanesulfonic acid); wk (week-old plant); and d (days-old plant). NR (data not reported in the original paper).

### 4.4. Fusion Procedures and Somatic Hybridization

**Figure 4:**
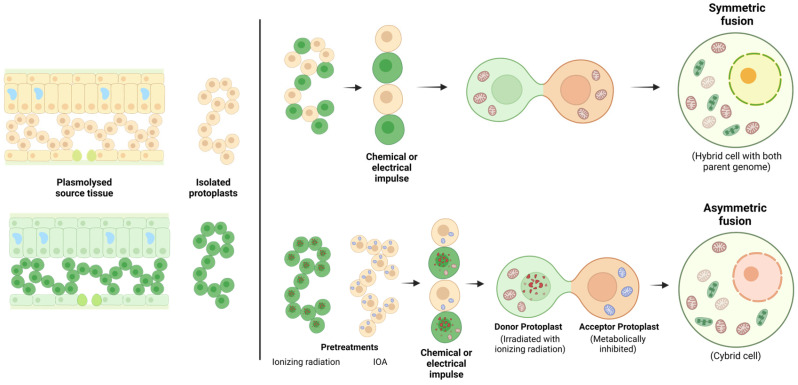
A simplified outline of symmetric and asymmetric protoplast fusion: donor protoplast (with red mitochondria) and acceptor protoplast (with blue mitochondria).

**Figure 5:**
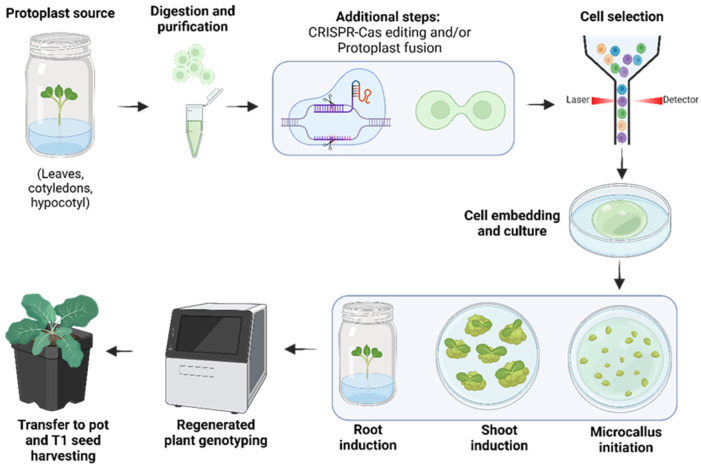
Overview of protoplast isolation and regeneration processes in *B. oleracea* cultivars, including key steps and genetic modification techniques.

Somatic hybridization offers a promising source of soma-clonal variation for in vitro breeding programs [123]. To enhance the development of intergeneric and interspecific hybrids in sexually incompatible species, somatic hybridization, which is an in vitro fusion of isolated protoplasts, is used to initiate a hybridization program. It allows the combination of desirable cytoplasmic and nuclear traits between Brassica species, genera, and tribes that are not normally achievable by conventional breeding. Somatic hybridization serves as a method to circumvent sexual incompatibility, enabling the creation of a wide range of somatic hybrids, including those that are intra- and interspecific, intergeneric, intertribal, and even interfamilial [85,123,124,125]. This technology has facilitated the creation of both intrageneric hybrids and intergeneric hybrids, as well as cybrids [126], facilitating the transfer of various desirable traits from parents to hybrids and cybrids [76,123,127,128]. Protoplast fusion has been successfully used to produce male-sterile lines that can be used for hybrid seed production on a commercial scale [16]. Two protoplasts can be fused even if they come from different species or incompatible varieties with the aim of regenerating novel germplasm [129]. The procedure involves four key phases: (i) isolating protoplasts from the parental species through the disruption of their cell walls; (ii) promoting the fusion of these protoplasts using electrical or chemical impulses; (iii) regenerating hybrid callus and whole plants from the fused protoplasts; and (iv) selecting and characterizing the somatic hybrid lines that exhibit desirable traits for further evaluation or use (Figure 4 and Figure 5). Typically, there exist two wide methods of protoplast fusion, which encompass chemical methods using binding substances such as polyethylene glycol (PEG) or dimethyl sulfoxide (DMSO) and physical methods such as electrofusion, which consists of the induction of depolarization of the protoplasts by means of high-frequency alternating currents [62,130,131]. This electric current generates contact between protoplasts and aligns them along the lines of the electric field generated, and with a direct current pulse, cell fusion is produced.

From amongst themselves, chemical fusion is the most widespread method used in *B. oleracea* L. breeding programs, and the chemical fusing agent mainly used in the Brassica genus is PEG-Ca^2+^ together with osmotic stabilizers such as mannitol or sorbitol (see References in Table 3). PEG acts as a molecular bridge and contributes to promoting adhesion and the formation of cytoplasmic bridges between protoplasts, ultimately leading to fusion.

Typically, a mixture of compounds such as sugar alcohols such as mannitol and sorbitol and salts including sodium chloride, calcium chloride, calcium nitrate, sodium hydroxide, and buffers is used [132]. Protoplast fusion can be classified into two categories, symmetric or asymmetric, based on how the genetic material (both nuclear and cytoplasmic) is contributed by the parent species. In symmetric fusion, both parental genomes contribute, since both parental nuclei fuse and become part of the nuclear genome in the resulting somatic hybrid equally (Figure 4). In asymmetric fusion, the genetic contribution from the donor is reduced because its nucleus is inactivated by radiation. As a result, the somatic hybrid contains the complete nuclear genome of the receptor parent, along with only fragments of the donor’s genome [2,62]. A cybrid, or cytoplasmic hybrid (Figure 4), is a singular type of hybrid cell, characterized by having its nuclear genome entirely derived from one parent, while the cytoplasmic components, including mitochondria and possibly other organelles, are contributed by both parents [76]. This results in a combination of nuclear DNA from one source and cytoplasmic elements from another, allowing for the study of interactions between nuclear and cytoplasmic genomes and the effects of different cytoplasmic environments on nuclear gene expression [133].

As discussed earlier, cybrids are generated by fusing protoplasts from donor cells, whose nuclei have been subjected to ionizing or non-ionizing radiation (Gamma rays, X-rays, or UV rays), with receptor protoplasts, in which the cytoplasmic organelles are metabolically inactivated using chemicals such as iodoacetamide (IOA) or iodoacetate (see references in Table 3). In the Brassicaceae family, the first successful report of protoplast hybridization amongst *B. campestris* and *B. oleracea* demonstrated that a 15 mM IOA treatment was necessary to produce 90% true hybrids. In contrast, a 7.5 mM IOA treatment was sufficient to inactivate *B. oleracea* protoplasts, but when fused with untreated *B. campestris* protoplasts, only 43% of true hybrids were achieved [134]. This outcome illustrated the “nurse effect”, where untreated protoplasts assist the treated ones in withstanding the higher IOA dose required for hybrid formation within 15 min. To counter this effect, Bruznican and colleagues extended from 20 to 25 min the exposure time while using 10 mM IOA [76]. This adjustment produced a fusion product with an intact donor nucleus and recipient cytoplasm. This cell fusion result is especially important in the creation of new CMS lines since it allows to overcome the sexual barrier by hybridizing the cytoplasmic donor variety/species, which is the source of sterility, with the nuclear genome of the other counterpart (Figure 4).

**Table 2:** Media used in vitro for plant regeneration from protoplast in the main *B. oleracea* cultivars.

Cultivars	Source	Medium Composition	Results	References
*B. oleracea* var. *italica*	Cotyledons and true leaves	Gamborg B5 [135] with vitamins, 2% (*w*/*v*) glucose, 7% (*w*/*v*) D-mannitol, 1 mg L^−1^ BAP, 1 mg L^−1^ NAA, and 1–0.25 mg L^−1^ 2,4-D. Liquid culture.	Protoplast division and microcallus formation	[80]
		½ Gamborg B5 [135] with vitamins, 2% (*w*/*v*) glucose, 4% (*w*/*v*) D-mannitol, 1 mg L^−1^ BAP, and 0.2 mg L^−1^ NAA. Liquid culture.		
		MS [136] micro- and macro-elements and vitamins, 3% (*w*/*v*) sucrose, 0.1–0.01 mg L^−1^ NAA, 1–3 mg L^−1^ BAP, and 0.8% agar.	Shoot induction	
		MS [136] micro- and macro-elements and vitamins, 3% sucrose, 0.1–0.01 mg L^−1^ NAA, 3 mg L^−1^ TDZ, and 0.8% agar.	Shoot inducing	
		½ MS [136] with vitamins, 2% sucrose, 0.1 mg L^−1^ NAA, and 0.8% agar.	Root inducing	
	True leaves	NH_4_NO_3_-free MS [136] micro- and macro-elements and vitamins, 60 g L^−1^ myo-inositol, 3% (*w*/*v*) sucrose, 2 mg L^−1^ 2,4-D, and 0.5 mg L^−1^ BAP, and pH 5.8. Liquid culture.	Protoplast division	[121]
		MS [136] micro- and macro-elements and vitamins, 100 mg L^−1^ myo-inositol, 30 g L^−1^ sucrose, 0.4 mg L^−1^ thiamin, 2 mg L^−1^ 2,4-D, 0.5 mg L^−1^ BAP, 0.4% (*w*/*v*) Gelrite and pH 5.8.	Microcallus formation	
		MS [136] micro- and macro-elements and vitamins, 100 mg L^−1^ myo-inositol, 30 g L^−1^ sucrose, 0.4 mg L^−1^ thiamin, 2 mg L^−1^ BAP, 0.5 mg L^−1^ NAA, 0.8% (*w*/*v*) plant agar, and pH 5.8.	Shoot induction	
		½ MS [136] micro- and macro-elements and vitamins, 2% sucrose, 0.8% plant agar, and pH 5.8.	Root induction	
	True leaves	Gamborg B5 [135] micro-, macro-elements with vitamins, 2% (*w*/*v*) glucose, 7% (*w*/*v*) D-mannitol, 1 mg L^−1^ NAA, 1 mg L^−1^ BAP, 0.25 mg L^−1^ 2,4-D, and pH 5.8. Liquid culture.	Protoplast division and microcallus formation	[106]
		MS [136] micro- and macro-elements with vitamins, 1% (*w*/*v*) sucrose, 2% (*w*/*v*) D-mannitol, 1 mg L^−1^ NAA, 1 mg L^−1^ IPA, 0.2 mg L^−1^ GA3, 0.8% (*w*/*v*) plant agar, and pH 5.8.	Shoot induction	
		MS [136] micro- and macro-elements with vitamins, 1% (*w*/*v*) sucrose, 0.1 mg L^−1^ NAA, 0.1 mg L^−1^ BAP, 0.6% (*w*/*v*) plant agar, and pH 5.8.	Root induction	
*B. oleracea* var. *capitata*	Etiolated hypocotyls	Kao and Michayluk [137] macro- and micro-elements and organic acids, Gamborg B5 [138] with vitamins, 74 g L^−1^ glucose, 250 mg L^−1^ casein enzymatic hydrolysate, 0.1 mg L^−1^ 2,4-D, 0.2 mg L^−1^ zeatin, and pH 5.6. Alginate layers embedding.	Protoplast division and microcallus formation	[73]
		MS [136] micro- and macro-elements with vitamins, 3% sucrose, 0.5 mg L^−1^ BAP, 0.2 mg L^−1^ NAA or 2 mg L^−1^ 2,4-D, 0.8% agar, and pH 5.8.	Shoot induction	
		MS [136] micro- and macro-elements with vitamins, 3% sucrose, 1 mg L^−1^ BAP, 2 mg L^−1^ 2,4-D, 0.8% agar, and pH 5.8.	Shoot induction	
	True leaves and hypocotyls	Kao and Michayluk [137] macro- and micro-elements and 0.4X organic acids, Gamborg B5 [138] vitamins, 2% (*w*/*v*) mannitol, 3% (*w*/*v*) sucrose, 250 mg L^−1^ casein enzymatic hydrolysate, 0.1 mg L^−1^ NAA, 0.2 mg L^−1^ zeatin, pH 5.6. Alginate layers embedding.	Protoplast division and microcallus formation	[84]
		MS [136] micro- and macro-elements and vitamins, 2% (*w*/*v*) sucrose, 0.25% (*w*/*v*) Phytagel, and pH 5.8.	Shoot and root induction	
		MS [136] micro- and macro-elements and vitamins, 0.4 mg L^−1^ calcium panthothenate, 0.1 mg L^−1^ GA3, 3.0 mg L^−1^ kinetin, and 3% (*w*/*v*) sucrose, 0.25% (*w*/*v*) Phytagel, and pH 5.8.	Shoot and root induction	
		MS [136] micro- and macro-elements, and modified vitamin composition, 0.5 mg L^−1^ nicotinic acid, 0.1 mg L^−1^ pyridoxine and thiamine, 3 mg L^−1^ glycine, and 2% (*w*/*v*) sucrose, 0.25% (*w*/*v*) Phytagel, and pH 5.8.	Shoot and root induction	
	Etiolated hypocotyls	Kao and Michayluk [137] macro- and micro-elements and 0.4X organic acids, Gamborg B5 [138] with vitamins, 20 g L^−1^ mannitol, 30 g L^−1^ sucrose, 250 mg L^−1^ casein enzymatic hydrolysate, 0.45 µM 2,4-D, 1 µM zeatin, and pH 5.6. Alginate layers embedding.	Protoplast division and microcallus formation	[82]
		MS [136] micro- and macro-elements with vitamins, 8.8 µM BAP, 2.7 µM NAA, 2% (*w*/*v*) sucrose, 2.5 g L^−1^ Gelrite, and pH 5.8.	Shoot induction	
		MS [136] micro- and macro-elements with vitamins, 2% (*w*/*v*) sucrose, 2.5 g L^−1^ Gelrite, and pH 5.8.	Root induction	
	Cotyledons and true leaves	Gamborg B5 [135] with vitamins, 2% glucose, 70 g L^−1^ D-Mannitol, 1 mg L^−1^ BAP, 1 mg L^−1^ NAA, and 1–0.25 mg L^−1^ 2,4-D. Liquid culture.	Protoplast division and microcallus formation	[80]
		½ Gamborg B5 [135] with vitamins, 2% glucose, 40 g L^−1^ D-Mannitol, 1 mg L^−1^ BAP, and 0.2 mg L^−1^ NAA.		
		MS [136] micro- and macro-elements with vitamins, 3% sucrose, 0.1–0.01 mg L^−1^ NAA, 1–3 mg L^−1^ BAP, and 0.8% agar.	Shoot inducing	
		MS [136] micro- and macro-elements with vitamins, 3% sucrose, 0.1–0.01 mg L^−1^ NAA, 3 mg L^−1^ TDZ, and 0.8% plant agar.	Shoot inducing	
		½ MS [136] micro- and macro-elements with vitamins, 2% sucrose, 0.1 mg L^−1^ NAA, and 0.8% (*w*/*v*) plant agar.	Root inducing	
	True leaves	Kao and Michayluk [137] macro- and micro-elements and organic acids, Gamborg B5 [138] vitamins, 250 mg L^−1^ casein hydrolysate, 74 g L^−1^ glucose, 0.1 mg L^−1^ 2,4-D and 0.2 mg L^−1^ zeatin, and pH 5.6. Alginate layers embedding.	Protoplast division and microcallus formation	[83]
		MS [136] micro- and macro-elements with vitamins, 750 mg L^−1^ CaCl_2_ 2H_2_O, 1 mg L^−1^ BAP, 0.1 µM PSK-α, 2% (*w*/*v*) sucrose, 0.25% (*w*/*v*) Gelrite, and pH 5.7–5.8.	Shoot and root formation	
		Gamborg B5 [135] micro-, macro-elements and vitamins without growth regulators, 750 mg L^−1^ CaCl_2_ 2H_2_O, 1 mg L^−1^ BAP, 0.1 µM PSK-α, 2% (*w*/*v*) sucrose, 0.25% (*w*/*v*) Gelrite, and pH 5.7–5.8.	Shoot and root formation	
*B. oleracea* var. *botytris*	Cotyledons and true leaves	Gamborg B5 [135] micro-, macro-elements with vitamins, 2% (*w*/*v*) glucose, 7% (*w*/*v*) D-Mannitol, 1 mg L^−1^ BAP, 1 mg L^−1^ NAA, 1–0.25 mg L^−1^ 2,4-D, and pH 5.6. Liquid culture.	Protoplast division and microcallus formation	[80]
		½ Gamborg B5 [135] with vitamins, 2% (*w*/*v*) glucose, 4% (*w*/*v*) D-Mannitol, 1 mg L^−1^ BAP, 0.2 mg L^−1^ NAA, and pH 5.6. Liquid culture.		
		MS [136] micro- and macro-elements with vitamins, 3% (*w*/*v*) sucrose, 0.1–0.01 mg L^−1^ NAA, 1–3 mg L^−1^ BAP, 0.8% (*w*/*v*) plant agar, and pH 5.8.	Shoot inducing	
		MS [136] micro- and macro-elements with vitamins, 3% (*w*/*v*) sucrose, 0.1–0.01 mg L^−1^ NAA, 3 mg L^−1^ TDZ, 0.8% (*w*/*v*) plant agar, and pH 5.8.	Shoot inducing	
		½ MS [136] micro- and macro-elements with vitamins, 3% (*w*/*v*) sucrose, 0.1 mg L^−1^ NAA, 0.8% (*w*/*v*) plant agar, and pH 5.8.	Root inducing	
	True leaves	Gamborg B5 [135] micro- and macro-elements with vitamins, 2% (*w*/*v*) glucose, 7% (*w*/*v*) D-Mannitol, 0.1 g L^−1^ MES, 1 mg L^−1^ NAA, 1 mg L^−1^ BAP, 0.25 mg L^−1^ 2,4-D, and pH 5.8. Agarose embedding.	Protoplast division and microcallus formation	[94,139]
		Gamborg B5 [135] micro- and macro-elements with vitamins, 2% (*w*/*v*) sucrose, 4% (*w*/*v*) D-Mannitol, 0.1 g L^−1^ MES, 1 mg L^−1^ NAA, 1 mg L^−1^ BAP, 0.25 mg L^−1^ 2,4-D, and pH 5.8. Agarose embedding.		
		MS [136] micro- and macro-elements with vitamins, 2% (*w*/*v*) sucrose, 1 mg L^−1^ 2iP, 1 mg L^−1^ NAA, 1 mg L^−1^ GA3, 0.6% (*w*/*v*) plant agar, and pH 5.8.	Shoot induction	
		MS [136] micro- and macro-elements with vitamins, 1% (*w*/*v*) sucrose, 0.1 mg L^−1^ BAP, 0.5 mg L^−1^ 2–4-D, 0.6% (*w*/*v*) plant agar, and pH 5.8.	Shoot induction	
		MS [136] micro- and macro-elements with vitamins, 1% (*w*/*v*) sucrose, 0.01 mg L^−1^ NAA, 0.6% (*w*/*v*) plant agar, and pH 5.8.	Root induction	

Abbreviations used: MS (Murashige and Skoog), KM (Kao and Michayluk), BAP (6-benzylaminopurine), NAA (α-naphthaleneacetic acid), 2,4-D (2,4-dichlorophenoxyacetic acid), TDZ (thidiazuron), IBA (Indole-3-butyric acid), PSK-α (Phytosulfokine-α), GA3 (gibberelic acid), MES (morpholinoethanesulphonic acid), 2iP (6-(γ,γ-Dimethylallylamino)Purine), and IPA (Indole-3-propionic acid).

Regarding this matter, Cardi and Earle [21] used the “Anand” cytoplasm derived from the wild species *B. tournefortii* to produce new CMS lines in *B. oleracea* with fertile oleracea cytoplasm and *B. rapa* with the sterile ‘Anand’ cytoplasm. The hybrid selection was carried out by dint of pretreatments of protoplasts with either iodoacetate or γ-rays, obtaining male-sterile cybrids with good flower morphology, attractiveness to insects, and female fertility. In a more recent study, Fujita et al. [140] established an alloplasmic *B. oleracea* CMS line with *Diplotaxis erucoides* cytoplasm, using alloplasmic *B. rapa* lines containing *D. erucoides* cytoplasm as a bridging plant and highlighted several advantageous traits of this CMS line. Importantly, cell hybridization through the protoplast fusion procedure can overcome sexual incompatibility barriers, enabling the use of wild species germplasm for the enhancement of crops and so on, to generate new cultivars with multiple agronomic benefits besides CMS. In this context, Sigareva and Earle [141] achieved transfer of the agriculturally important traits as resistance to *Alternaria brassicicola* to *B. oleracea* from *Capsella bursa-pastoris*, which cannot naturally cross with cultivated cruciferous crops, can be successfully hybridized with them using protoplast fusion. In a study by Wang et al. [85], an interspecific asymmetric somatic hybrid was created between *B. oleracea* var. *botrytis* and *B. nigra* to transfer disease-resistance genes from the wild species into *B. oleracea* var. *capitata*. The regenerated plants demonstrated a high level of resistance to black rot, indicating that the disease-resistant genes from *B. nigra* had been successfully incorporated into the cultivated variety [85]. Likewise, *Camelina sativa* was used to introduce resistance to blackleg into *B. oleracea* var. *capitata* through protoplast fusion [142]. By applying asymmetric somatic hybridization in combination with a disease selection process, resistance traits against *Phoma lingam* and turnip mosaic virus were successfully transferred from *B. juncea* into different *Brassica oleracea* cultivars [138,143]. Other research has shown that somatic hybrids typically display more genetic diversity compared to their parental lines, which aids in the generation of new genetic resources (reviewed by [87]). Therefore, protoplast fusion proves to be an effective and efficient method for introducing genetic material from wild species into cultivated crops, making it a valuable tool for developing new varieties with improved ploidy levels, better disease resistance, and enhanced agronomic and quality traits.

### 4.5. Genome and Gene Editing

Genetic transformation is the process by which a cell of one genotype assimilates DNA from another cell with a different genotype present in its environment, leading to changes in its own genotype and gene expression [115]. This mechanism, known as genetic transformation, involves the uptake of homologous or heterologous DNA by a recipient cell, which can occur naturally or be artificially induced. The DNA may be derived from natural sources or synthesized, and its incorporation into the recipient cell facilitates horizontal gene transfer and expression [144]. Genetic transformation is classified into two main categories: natural and artificial. Natural transformation is a process that occurs in traditional breeding strategies, such as backcrossing and selection, used to improve various crop traits but is slow and resource-intensive [145]. However, in order to speed up this process, artificial genetic transformations have been used. Routinely, *Agrobacterium*-mediated genetic transformation has been used in plant transformation [116]. This technique entails isolating fragments of the target gene and constructing a vector to transport these fragments. Subsequently, *Agrobacterium* in a competent state is employed to facilitate the transfer of the vector into plant cells. While *Agrobacterium*-mediated transformation is a widely used and effective method for creating genetically modified plants, it does have several disadvantages and limitations, among which highlights the random integration of the T-DNA into the plant genome since it occurs randomly and can disrupt important genes or regulatory elements, leading to unintended genetic changes or undesirable phenotypes [146,147]. Notwithstanding some of the drawbacks of *Agrobacterium*-mediated transformation, this technique has been widely used in Brassica breeding throughout protoplast technology [117,144].

However, a new breeding technique called CRISPR/Cas (clustered regularly interspaced short palindromic repeats-associated protein–Cas) has the potential to accurately and rapidly improve many traits related to quality, yield, nutrition, and abiotic and biotic stress tolerance in crops [120]. CRISPR/Cas is a prokaryotic adaptive defense system that protects bacteria and archaea from invading genetic materials such as viruses or plasmids [148]. These systems evolve rapidly, resulting in significant structural and functional diversity. Progress in the study of CRISPR/Cas defense systems has resulted in the creation of CRISPR/Cas9 and CRISPR/Cas12, both of which are RNA-guided genome editing technologies. [120,149,150]. Due to their simplicity, high efficiency, versatility, and ability to target multiple genes simultaneously, CRISPR/Cas technologies have been widely adopted, revolutionizing all areas of molecular biology [151,152]. The CRISPR/Cas9 technology, commonly known as a programmable molecular scissor, is an advanced RNA-guided genome-editing tool that enables the precise introduction of mutations into any sequenced genome [153]. Initially, it was employed for genetic engineering in plants [154] since CRISPR/Cas9 has shown great promise in crop improvement, enhancing important agronomic traits such as postharvest shelf life, an increase in important nutritional metabolites such as glucoraphanin accumulation, and biotic and abiotic stress resistance, among other factors [23,155,156]. There are two main approaches for regenerating plants from protoplasts edited using CRISPR; one involves transfecting the protoplasts with plasmid DNA, while the other entails using a preassembled Cas-gRNA complex, which is known as a ribonucleoprotein (RNP). Nonetheless, when protoplasts are transiently transfected with DNA, a considerable proportion of the regenerated plants show unintended insertions from the CRISPR plasmid [157,158]. RNPs mitigate the risk of plasmid DNA integration into the plant genome, as no foreign DNA is introduced during the transfection process [159]. These results underscore the potential and feasibility of utilizing isolated protoplasts for CRISPR-based gene editing, especially in crops with long juvenile phases, those that are heterozygous, or those that are propagated asexually.

**Table 3:** Development of somatic hybrids with valuable agronomic traits through protoplast fusion in *B. oleracea* cultivars.

Parental Combinations	Type of Cross	Tissue Source	Pre-Treatment	Fusion Method	Fusion Type	Hybrid Performance	References
*B. oleracea* var. *capitata* × *B. juncea* *B. oleracea* var. *capitata* × *B. rapa* *B. oleracea* var. *capitata* × *B. juncea*	Interspecific	Hypocotyl and cotyledons	2 mM IOA 15 min at 4 °C.	50% (*w*/*v*) polyethylene glycol (PEG 6000).	Symmetric	Production of sterile hybrids with cold tolerance and atrazine resistance.	[143]
*B. oleracea* var.* botrytis × B. rapa*	Interspecific	Leaves	X-ray irradiation of 92 Gy and 2 mM IOA for 15 min at 4 °C.	50% (*w*/*v*) polyethylene glycol (PEG 6000).	Asymmetric	Cold tolerance and Ogura male sterile hybrids.	[160]
*B. oleracea* var.* capitata × A. thaliana*	Intergeneric	Hypocotyls and leaves	2 mM of IOA for 15 min at 25 °C and UV radiation at 4680 J m^−2^.	50% (*w*/*v*) polyethylene glycol (PEG 6000).	Asymmetric	Establishment of cabbage lines with CMS (male sterile hybrid production).	[161]
*B. oleracea* var.* capitata × B. nigra*	Interspecific	Cotyledons and hypocotyls	0.0875 J cm^−2^ UV irradiation.	40% (*w*/*v*) polyethylene glycol (PEG 1500).	Symmetric	Resistance to *Xanthomonas campestris* pv. *campestris*	[162]
*B. oleracea* var.* botrytis × Matthiola incana*	Intergeneric	Hypocotyls and leaves	None.	40% (*w*/*v*) polyethylene glycol (PEG 1500).	Symmetric	α-linolenic acid increases content and aphid resistance.	[128]
*B. oleracea* var.* italica × B. campestris*	Interspecific	Cotyledons and hypocotyls	None.	40% (*w*/*v*) polyethylene glycol (PEG 1450) and dimethyl sulfoxide (DMSO).	Symmetric	Bolting resistance.	[142]
*B. oleracea* var.* capitata × B. rapa*	Interspecific	Leaves	3 mM IOA 15 min at 4 °C.	40% (*w*/*v*) polyethylene glycol (PEG 3350).	Symmetric	Production of interspecific hybrids resistant to *Erwinia carotovora* subsp. *Carotovora.*	[59]
*B. oleracea* var. *capitata × B. oleracea* var. *botrytis* *B. oleracea* × *B. nigra* *B. oleracea* × *Diplotaxis tenuifolia* *B. oleracea* × *Matthiola incana*	Intraspecific Interspecific Interspecific Interspecific	Hypocotyls and leaves	X-ray irradiation of 92 Gy and 3 mM IOA for 15 min at 4 °C.	12.5% (*w*/*v*) polyethylene glycol (PEG 6000).	Asymmetric	Production of interspecific hybrids resistant to *Alternaria* spp.	[132]
*B. oleracea* var.* botrytis × B. nigra*	Interspecific	Hypocotyls and leaves	0.0875 J cm^−2^ UV irradiation and 2 mM IOA for 15 min at 4 °C.	40% (*w*/*v*) polyethylene glycol (PEG 1500).	Asymmetric	Resistance to black rot.	[85]
*B. oleracea* var.* italica × B. juncea*	Interspecific	Cotyledons and hypocotyls	None.	40% (*w*/*v*) polyethylene glycol (PEG 1450).	Symmetric	Resistance to turnip mosaic virus.	[163]
*B. oleracea* var. *capitata × C. bursa-pastoris*	Intergeneric	Leaves	3 mM IOA for 15 min at 4 °C.	50% (*w*/*v*) polyethylene glycol (PEG 6000).	Symmetric	Resistance to *Alternaria brassicicola*.	[122]

Abbreviations used: IOA (iodoacetamide); PEG (polyethilenglycol).

By utilizing protoplasts in combination with a CRISPR-Cas9 RNP complex, gene editing can be achieved directly in the T0 generation without the need to incorporate foreign CRISPR DNA constructs. The RNP complex consists solely of the Cas9 protein and guide RNA (gRNA), which are preassembled and introduced directly into the protoplasts. This method avoids the integration of foreign DNA into the plant genome, as the gene editing process occurs through the action of the protein and RNA only, without the need for plasmid DNA or other recombinant DNA constructs. As a result, edited plants are produced free of foreign DNA sequences. Moreover, this approach bypasses the need for hybridization, introgression, or back-crossing of progeny. Because the regenerated plants stem from an isolated edited protoplast, all cells share the same genetic background, assuring that the edited alleles are reliably passed down to subsequent generations [129]. The CRISPR-Cas9 system has become a crucial tool for accelerating the development of novel germplasm with targeted traits, benefiting from the vast insights provided by whole-genome sequencing and functional genomics. However, genetic modification of *B. oleracea* L. has proven more challenging compared to other cruciferous species like *B. juncea* and *B. napus*, leading to fewer studies focused on *B. oleracea*. Ma et al. [164] demonstrated effective genome editing in cabbage cells by establishing stable expression of the CRISPR/Cas9 system. Their work resulted in the knockout of the phytoene desaturase gene (BoPDS), along with its paralog, using multiple independent sgRNA-expression cassettes. The knockout of BoPDS caused an albino phenotype in transgenic plants. Furthermore, Chinese cabbage exhibits sensitivity to low temperatures during seed germination, which can occasionally lead to bolting (early onset of flowering) in the fall. [165,166]. This phenomenon leads to reduced crown quality and yield losses for crops. Other authors [167] applied a multiplex CRISPR/Cas9 system to precisely edit the BrVRN1 gene, which serves as an important transcriptional repressor that triggers the transition to flowering in response to extended cold periods. This mutagenesis resulted in the development of a *B. rapa* strain train with a delayed flowering trait, with the BrVRN1 gene identified as a key positive regulator of flower induction in the plant. Otherwise, Jeong and colleagues achieved an early flowering phenotype in Chinese cabbage that was independent of vernalization through genome editing using CRISPR/Cas9 technology on isolated protoplasts [168]. Furthermore, Kim et al. [121] developed glucoraphanin (GR)-rich broccoli using CRISPR/Cas9 technology to edit the flanking sequence of the 9 bp deleted MYB28 gene in broccoli protoplasts. By introgressing the MYB28 allele from the wild relative *B. villosa* into *B. oleracea* var. *italica*, they increased GR levels, resulting in one amino acid substitution and the deletion of three amino acids. These findings indicate that the same mutation in the BolMYB28 gene may be applicable to other crops within the same genus, including cabbage, cauliflower, and kale, facilitating the development of high-GR varieties.

Simultaneously, Neequaye et al. [169] knocked out the MYB28 gene using CRISPR/Cas in broccoli protoplasts, which resulted in the downregulation of glucosinolate biosynthesis genes with the subsequent reduction in the accumulation of glucoraphanin in the leaves and florets of field-grown MYB28 mutant *B. oleracea* var. *oleracea* plants. However, the accumulation of sulfate, S-methyl cysteine sulfoxide, and indole glucosinolate in leaf and floret tissues remained unchanged. In a separate study, Ma et al. [164] employed a CRISPR/Cas9 gene editing system utilizing endogenous tRNA processing to accomplish highly efficient and heritable mutagenesis in *B. oleracea* var. *Capitata*. These authors achieved multisite and multiple gene mutations by employing a construct with tandemly arrayed tRNA-sgRNA architecture, enabling the expression of multiple sgRNAs targeting the BoPDS, the male-sterility-associated gene (BoMS1), the S-receptor kinase gene (BoSRK), and the mutation of the BoSRK3 gene completely eliminated self-incompatibility, transforming the previously self-incompatible line into a self-compatible one. Furthermore, the mutation of the BoMS1 gene led to a completely male-sterile mutant, which displayed high cross-compatibility with its non-mutant isoline during flowering. This fact was made possible by the concurrent mutation of the BoSRK3 gene. Altogether, these genetic modifications facilitated the cost-effective propagation of the male-sterile cabbage line through bee-mediated cross-pollination.

## 5. Perspectives in Brassicaceae Breeding

Due to its significant utility in plant breeding, the CMS phenotype is often introduced into target crops from natural populations or developed anew in laboratory settings. It can be experimentally induced through intraspecific, interspecific, or intergeneric crosses, as well as through protoplast fusions or genetic engineering [170,171]. The composition of the nuclear genome makes cybrids particularly appealing for breeding programs, as the nuclear genome is entirely derived from one parent ensures the cultivar’s genetic integrity [172,173]. Moreover, cytoplasmic hybrids commonly exhibit CMS, making them a valuable asset in plant breeding efforts. Somatic hybridization serves as a powerful method for transferring genomes or genomic fragments from wild plants with desirable agronomic characteristics into commercial crops [174]. Protoplast fusion effectively addresses the challenges posed by pre- and post-zygotic barriers in sexual hybridization, allowing for the combination of sexually incompatible germplasms from different crops and even from phylogenetically distant plants [51,122,150]. This method not only allows for the integration of desirable traits from uncultivated varieties but also facilitates the transfer of traits encoded by plastid or mitochondrial genomes into commercial crops. For instance, in the Brassica genus, somatic hybridization has successfully introduced valuable mitochondrial-encoded traits, as outlined in Table 3. As stated above, these traits include resistance to multiple diseases caused by fungi and viruses, enhanced tolerance to cold temperatures, improved bolting resistance, and the generation of hybrids demonstrating CMS performance. Moreover, somatic hybridization offers the advantage of replacing the cytoplasm of a cultivar in a single step, making it a highly efficient method compared to traditional breeding techniques, which typically require several backcrosses to achieve similar outcomes. This efficiency is particularly beneficial for breeders looking to quickly incorporate valuable traits into existing cultivars.

In Europe, CMS varieties are generally exempt from strict genetically modified organisms (GMO) regulations, as they do not involve foreign DNA insertion via recombinant DNA technology, a key criterion for GMOs. Organic production is governed by European standards (Regulation (EU) 2018/848), which sets rules for organic products without restricting *B. oleracea* L. CMS varieties. However, some organic farming customers prefer to avoid CMS varieties derived from protoplast fusion, as these are not produced under natural conditions. Despite this, CMS varieties are widely accepted in European agriculture with no significant regulatory restrictions. Given this context, Brassicaceae breeding is expected to blend traditional approaches commonly used today with advancements in biotechnology to accelerate the development of market-ready cultivars. Breeding companies are likely to invest in cutting-edge breeding technologies based on the crop’s significance, ensuring a profitable return on their investments. This is especially pertinent for *B. oleracea* species, which are considered highly important due to their high economic and nutritional value [6,175]. In the past decade, the field of *B. oleracea* genetics and genomics has made remarkable strides, with detailed gene function studies illuminating the mechanisms behind key breeding traits. Liu et al. [176] published the first report of the draft genome of cabbage line 02–12, which is recognized for its outstanding agronomic qualities. That same year, Parkin et al. [177] published the draft genome of TO1000, a doubled haploid kale-like variety. Both genomes were assembled using next-generation sequencing technology. More recently, advancements in third-generation sequencing have enabled the completion of genome assemblies and the generation of high-quality genomes for various cabbage lines with distinct morphologies as well as for broccoli and cauliflower [178,179,180]. The forthcoming objective will be to effectively translate this genomic knowledge into practical breeding strategies. This could involve developing molecular markers to assist in the selection of pre-breeding materials or employing genetic engineering techniques to introduce specific traits into existing cultivars. By bridging the gap between genomic research and practical application, breeders can enhance the efficiency of breeding programs and accelerate the development of improved varieties.

In *B. oleracea* L. breeding, profitable, well-established methods of CMS introgression through asymmetric protoplast fusion or sexual crossings with distantly related species have been employed, although researchers should also concentrate on gaining a deeper understanding of the mechanisms by which mitochondria contribute to this trait. By elucidating these underlying processes, it may be possible to enhance the stability and effectiveness of CMS lines in breeding programs. One of the most significant threats in *B. oleracea* breeding is the increasing prevalence of diseases such as black rot, which is caused by the Gram-negative bacterium *X. campestris* pv. *campestris* (Xcc), clubroot disease caused by the protist pathogen *Plasmodiophora brassicae*, and the Turnip yellows virus. To combat these hazards, developing cultivars with greater tolerance to biotic stress must be a continuous priority in *B. oleracea* breeding. This is especially important as pathogen effectors evolve to evade the plant’s innate immune responses, leading to increased susceptibility over time. Numerous reports have documented the rise in these diseases as a result of interspecific hybridization (see references in Table 3). In contemporary agricultural practices, there is a strong emphasis on reducing pesticide use and promoting organic farming methods, which underscores the importance of breeding disease-resistant cultivars. Such cultivars would not only enhance crop resilience but also align with sustainable agriculture goals, benefiting both growers and the environment by minimizing the reliance on chemical treatments.

Addressing the future of *B. oleracea* breeding requires acknowledging the urgent reality of climate change: our environment is shifting, and Brassicaceae may find it challenging to adapt. In the coming years, we can expect rising temperatures, extended droughts, and increased CO_2_ emissions [181]. Prolonged drought periods can drastically affect *B. oleracea* yields by extending the growing season and compromising the quality of the crowns [12]. Currently, the primary approach involves implementing stricter conditions in selection trials [182], such as reduced irrigation, rather than developing cultivars capable of withstanding these climate-related stresses [183,184]. Breeders and scientists must confront the challenges posed by climate change by developing cultivars that can withstand the extreme environmental conditions associated with global warming. With traditional breeding cycles lasting between 8 and 15 years, there is an urgent need to accelerate progress [52]. Alternative breeding techniques, such as genome editing with CRISPR-Cas9, offer promising avenues for speeding up the development of resilient cultivars. Additionally, incorporating CMS technology can further enhance *B. oleracea* breeding by speeding up hybrid seed production and improving the efficiency of crossing programs [138]. Incorporating CMS technology allows breeders to create hybrids more rapidly and efficiently, which is essential in adapting to climate change pressures. This technique helps overcome the limitations of traditional breeding methods by enabling the development of high-yielding hybrids without the need for extensive backcrossing. By combining CMS with genome editing, breeders can not only improve yield and resilience but also introduce traits such as disease resistance, drought tolerance, and nutrient enrichment more effectively. Moreover, efforts should be directed toward conserving genetic resources and expanding the gene pool by integrating more wild accessions and enhancing genetic diversity and resilience against environmental stressors.

## 6. Concluding Remarks

CMS traits from wild plants have been successfully transferred to agriculturally important species through somatic hybridization, which involves the fusion of different types of plant cells. Somatic hybrids can also be generated to create CMS de novo by recombining the mitochondrial genomes of two parent plants or by dissociating the CMS cytoplasm from the nuclear Rf alleles. This positions somatic hybridization as a particularly efficient approach for incorporating CMS into breeding programs. The application of CMS technology enables breeders to rapidly and effectively produce hybrids, which is vital for adapting to the challenges presented by climate change. This technique overcomes the limitations of traditional breeding methods by allowing the development of high-yielding hybrids without extensive backcrossing. By combining CMS with advanced techniques like CRISPR-Cas9 genome editing, breeders can improve yield and resilience while introducing traits such as disease resistance, drought tolerance, and nutrient enrichment more effectively. The speed and accuracy of CRISPR-Cas9 make it an invaluable tool for developing cultivars that can meet the demands of a changing climate and evolving consumer preferences. According to the above-mentioned, the development and application of robust plant regeneration protocols are essential for modern breeding programs. Plant regeneration from somatic cells or tissues through tissue culture techniques is a critical step for successful genetic transformation and genome editing. Efficient regeneration protocols ensure that genetically modified plants can be produced in sufficient quantities and with high fidelity to the desired traits. In conclusion, this review underscores the importance of integrating CMS technology, protoplast fusion and de novo plantlet regeneration, and CRISPR-Cas9 genome editing in *B. oleracea* breeding (Figure 5). These advanced biotechnological tools hold great promise for developing high-performing, resilient crop varieties that can meet the challenges of modern agriculture and contribute to sustainable food production.
